# Case 1/2018 - Young Male with Heart Disease Expressed Mainly as Ventricular Arrhythmia, Right Ventricular Dysfunction and Syncope

**DOI:** 10.5935/abc.20180031

**Published:** 2018-02

**Authors:** Desiderio Favarato, Luiz Alberto Benvenuti

**Affiliations:** Instituto do Coração (InCor) HC-FMUSP, São Paulo, SP - Brazil

**Keywords:** Arrhythmias, Cardiac, Ventricular Dysfunction, Right, Syncope, Diagnostic Imaging, Defibrillators, Implantable, Catheter Ablation, Heart Transplantation

The patient is a 36-year-old male with history of syncope since the age of 20 years and dyspnea since the age of 30 years. He reported marked worsening of those symptoms in the preceding 6 months from the last admission.

His symptoms began with episodes of syncope at the age of 20 years. On the investigation, a neurological cause was ruled out, and heart disease with cardiac dilatation and systolic dysfunction of the left ventricle was detected. The patient denied dyspnea at that time.

On ECG (2001), low-voltage complexes in the frontal plane and final conduction delay were observed.

The laboratory tests (April 2001) revealed: hemoglobin, 14.6 g/dL; hematocrit, 42%; leukocytes, 4900/mm^3^ (31% neutrophils, 14% eosinophils, 1% basophils, 37% lymphocytes and 17% monocytes); platelets, 170000/mm^3^; potassium, 4 mEq/L; sodium, 134 mEq/L; creatinine, 1 mg/dL; urea, 39 mg/dL; PT(INR), 1.0; aPTT (rel. times), 1.16.

Magnetic resonance angiography (April 12, 2001) of the internal carotid, anterior, middle and posterior cerebral and vertebral arteries was normal.

The echocardiogram (June 5, 2001) revealed the following: diameters of the aorta and left atrium, 32 mm, and of the left ventricle (diastolic/systolic), 65/52 mm; ejection fraction (Simpson), 40%; septal and posterior wall thickness, 10 mm; right ventricle, 23 mm. In addition, there were indirect signs of pulmonary hypertension, estimated as 40 mmHg. The left ventricle showed diffuse hypokinesia, and there was marked calcification of the mitral valve leaflets.

The exercise test (July 2, 2001) up to 70% of the predicted maximal heart rate showed no change suggestive of ischemia. The blood pressure curve had a depressed pattern. Isolated and paired ventricular extrasystoles were frequent, as were short-duration nonsustained ventricular tachycardia episodes. The test was interrupted because of exhaustion.

The management consisted in heart failure treatment and cardioverter defibrillator implantation, the cardiac findings being attributed to sequelae of myocarditis. His daily prescription was as follows: losartan 50 mg, amiodarone 400 mg, spironolactone 25 mg, and metoprolol succinate 75 mg.

His laboratory tests (Dec 2002) showed: cholesterol, 167 mg/dL; TSH, 6.8 µg/mL; free T4, 1.6 ng/mL; triglycerides, 43 mg/dL; glycemia, 90 mg/dL; uric acid, 4.3 mg/dL.

His new echocardiogram (March 2004) revealed the following diameters: aorta, 31 mm; left atrium, 34 mm; left ventricle (diastolic/systolic), 60/50 mm. His ejection fraction was 42%, and the septal and posterior wall thickness, 7 mm. The left ventricle was dilated and diffusely hypokinetic. The right ventricle was 31-mm thick, dilated and hypokinetic. The right atrium was dilated. Pacemaker electrodes were identified in the right chambers. Neither atrioventricular nor ventriculo-arterial valves had changes.

The patient developed dyspnea on maximal exertion, but no syncope. There was an episode of inappropriate shock from the cardioverter defibrillator in 2005, due to problems in the ventricular electrode, which was replaced. The disease course was uneventful until 2012.

Cardiopulmonary exercise test showed variation of heart rate from 57 bpm to 122 bpm, and of blood pressure from 120/80 mm Hg to 185/70 mm Hg, and maximal oxygen consumption of 28.4 mL/kg/min.

During that time, the echocardiography showed ejection fraction ranging from 44% (2001) to 39% in 2006, and of 28% in 2011, while the diastolic diameter of the left ventricle remained constant (65 mm in 2001, 66 mm in 2006 and 2011).

The patient was referred for surgical treatment assessment for heart failure, but, because he had few symptoms and good physical capacity, clinical and pharmacologic management was maintained (2012).

Computed tomographic angiography of the coronary arteries (February 5, 2014) showed neither calcifications nor obstructive lesions.

In February 2014, the cardioverter defibrillator delivered a shock, and the patient underwent radiofrequency ablation of the arrhythmia.

During the electrophysiological study (February 3, 2014), atrial stimulation triggered atrial fibrillation, which organized as flutter arising from the cavotricuspid isthmus, which was blocked. On the electrical mapping of the right ventricle, areas of scar and low-voltage late potentials were observed in the basal posterior and lateral walls, and radiofrequency pulses were applied, eliminating them.

During left ventricular epicardial mapping, a non-dense scar area was identified in the basal portion of the posterolateral wall, as were late potentials. No radiofrequency pulse was applied to those sites.

During the study, poorly-tolerated sustained ventricular tachycardia of epicardial origin was observed and reversed with electrical cardioversion. The procedure was successful.

In March 2014, the patient sought the emergency unit reporting three shocks of the implantable cardioverter defibrillator in the morning while walking on the beach, the first being preceded by tachycardia. The patient had bradycardia (40 bpm) while receiving intravenous amiodarone. He remained asymptomatic during hospitalization (March 3 to 14, 2014). The patient was discharged with the following prescription: 50 mg of losartan, 200 mg of amiodarone, 25 mg of spironolactone and 75 mg of metoprolol succinate, in addition to programming of the implantable cardioverter defibrillator to pacemaker capture threshold of 40 bpm.

His catheterization (September 29, 2015) revealed: mean right atrial pressure, 14 mm Hg; right ventricular pressures (systolic/initial diastolic/final diastolic), 28/06/14 mmHg; pulmonary artery pressures (systolic/diastolic/mean), 28/18/21 mmHg; pulmonary occlusion pressure, 18 mmHg; aortic pressures (systolic/diastolic/mean), 93/60/71 mmHg; cardiac output, 3.78 mL/min; pulmonary vascular resistance, 0.79 woods; arterial O_2_ saturation, 99.1%; venous O_2_ saturation, 70.4%.

A new electrophysiological study was performed (September 29, 2015). At the beginning of the procedure, the patient was in sinus rhythm, and had periods of atrioventricular block 2:1 and total atrioventricular block during the procedure. To desfibrillitor was summed a pacemaker elecatrode programmed to VVI pacing for 40 bpm. Electrophysiological mapping and voltage mapping of the right ventricle (endocardial) were performed, evidencing a scar area in the lateral region of the right ventricular outflow tract, extending to the tricuspid annulus. The extra stimuli induced type I ventricular tachycardia (VT1) with positive complexes in I and aVL leads, with negative superior axis in V_1_ and no transition. The activation mapping during tachycardia evidenced mesodiastolic potential in the scar area of the lateral region of the right ventricular outflow tract. Radiofrequency application in that site terminated the VT1. In addition, the scar area was homogenized from the lesion to the tricuspid annulus. New tests with extra stimuli failed to induce arrhythmias.

The patient had no arrhythmia and only a few symptoms of dyspnea.

During a medical visit in January, 2016, the patient reported worsening of symptoms, with dyspnea occurring while taking a bath or walking less than two blocks, in addition to weight loss, although his appetite was preserved. His daily prescription was as follows: amiodarone 400 mg, spironolactone 25 mg, metoprolol succinate 50 mg, losartan 50 mg, levothyroxine 75 mcg, magnesium 400 mg, and furosemide 20 mg.

His physical examination revealed blood pressure of 100/80 mm Hg, heart rate of 60 bpm, regular perfusion, and signs of neither hypervolemia nor pulmonary congestion. His daily dose of losartan was increased to 75 mg.

The patient continued very limited regarding his daily activities, being placed on the waiting list for cardiac transplantation, which was performed on April 12th, 2016.

## Clinical aspects

The patient had syncope episodes since the age of 20 years, and heart failure since the age of 30 years, undergoing cardiac transplantation at the age of 36 years.

Since symptom onset, heart disease with marked left ventricular dilatation and moderate dysfunction was detected. The exercise test revealed frequent ventricular arrhythmia. His syncope episodes were attributed to malignant ventricular arrhythmias, and a cardioverter defibrillator was implanted.

The patient remained stable and with no syncope episode for 11 years, when marked decrease in ventricular ejection fraction was detected.

At the age of 34 years, the cardioverter defibrillator delivered an appropriate shock during an episode of ventricular tachycardia, and the patient was submitted to an electrophysiological study, which triggered atrial fibrillation and the cavotricuspid isthmus was blocked. During the same procedure, areas compatible with scars and low-voltage late potentials in the basal posterior and lateral walls of the right ventricle were observed, undergoing ablation, which eliminated the potentials. However, during the procedure, poorly-tolerated sustained ventricular tachycardia of epicardial origin was observed and reversed with electrical cardioversion.

One year later, the patient had a new episode of tachycardia and appropriate cardioverter defibrillator discharge, undergoing then a new electrophysiological study, which evidenced a scar in the right ventricular outflow tract, extending to the tricuspid annulus. The extra stimuli triggered VT1 arising in the right ventricle, which was terminated with ablation with radiofrequency application.

A few months later, the patient was hospitalized due to NYHA functional class III heart failure, being placed on the waiting list for cardiac transplantation.

This is a case of heart disease, presenting as episodes of syncope due to ventricular arrhythmias and mild left ventricular dysfunction, despite left ventricular dilatation.

Some of the heart diseases that progress mainly with ventricular arrhythmias are as follows: Chagas heart disease, sarcoidosis, hypertrophic cardiomyopathy, and arrhythmogenic right ventricular cardiomyopathy.

Regarding Chagas disease, it is known to cause frequent arrhythmias, heart failure and sudden death, but the patient had neither a typical ECG nor a typical echocardiogram. His ECG showed neither right bundle branch block nor left anterior hemiblock, and his echocardiogram revealed neither diffuse marked hypokinesia nor an apical aneurysm. In addition, apparently there was neither positive epidemiology for that disease, nor predominance of right heart failure signs.^1,2^

In sarcoidosis, heart impairment is mainly characterized by atrioventricular blocks, malignant arrhythmias and sudden death, all caused by infiltration of the conduction system and myocardium by noncaseous granuloma. Some studies on pulmonary or systemic sarcoidosis have reported cardiac impairment in 5% of the patients and in up to 25% of postmortem examinations. However, imaging tests have shown impairment in up to 50% of the patients. Individuals with extracardiac sarcoidosis diagnosis confirmed on biopsy should be asked about the symptoms of syncope, presyncope and heart palpitations. The ECG is mandatory in all patients with sarcoidosis, and if any abnormality is found, echocardiography and other imaging tests, such as magnetic resonance imaging and 14-fluorodeoxyglucose PET, can be useful.^3^

The present case could be perfectly diagnosed as sarcoidosis, except for the lack of extracardiac sarcoidosis findings, mainly pulmonary impairment, which is the most common finding.

Hypertrophic cardiomyopathy can cause syncope and sudden death. However, our patient showed no cardiac hypertrophy with at least one wall with minimal thickness of 15 mm.^4^

Arrhythmogenic ventricular cardiomyopathy is a genetic disease due to mutations in the genes encoding desmosine, characterized by fibrofatty infiltration of the right ventricular myocardium. The changes can begin in three regions of the right ventricle: ventricular inlet, outflow tract and tip.

The diagnostic criteria of arrhythmogenic ventricular cardiomyopathy were reviewed by a Task Force in 2010. They comprise electrocardiographic, echocardiographic, magnetic resonance imaging and right ventriculographic findings, family history, and histologic changes on endomyocardial biopsy.

On ECG at rest, the major criteria are: T-wave inversion in V_1_ to V_3_ in individuals aged over 14 years, in absence of right bundle branch block (QRS ≥ 120 ms), epsilon waves at the end of the QRS complex in V_1_ to V_2_. The minor criteria are: T-wave inversion in V_1_ and V_2_, in absence of right bundle branch block, or in V_4_ to V_6_; or T-wave inversion in V_1_ to V_4_ at that same age group with right bundle branch block, high-resolution ECG lasting ≥ 114 ms, and low-voltage late potentials (< 40 µV), at the end of the QRS complex, > 38 ms.

Regarding the presence of arrhythmia, a major criterion is the occurrence of sustained or nonsustained ventricular tachycardia with morphology of left bundle branch block with superior axis. The minor criteria are: ventricular tachycardia with QRS morphology of left bundle branch block with inferior axis or frequent ventricular extrasystoles > 500/24 hours.

On two-dimensional echocardiography, the major criteria are regional right ventricular akinesia, dyskinesia or aneurysm, accompanied by one of the following changes: ventricular outflow tract dilatation (≥ 32 mm) or with correction for body surface ≥ 19mm/m^2^ in parasternal long-axis view; ≥ 26 mm or with correction for body surface ≥ 21 mm/m^2^ in parasternal short-axis view or ejection fraction ≤ 33%. The minor criteria are: regional right ventricular akinesia or dyskinesia and one of the following changes: ventricular outflow tract dilatation ≥ 29 and < 32 mm or with correction for body surface ≥ 16 and < 19 mm/m^2^ in parasternal long-axis view; or ≥ 32 and < 36 mm in parasternal short-axis view or with correction for body surface ≥ 18 and < 21 mm/m^2^; or ejection fraction > 33% and ≤ 40%.

On magnetic resonance imaging, the major criteria are akinesia or dyskinesia or dyssynchronous right ventricular contraction, in addition to one of the following changes: right ventricular end-diastolic index ≥ 110 mL/m^2^ (male) and ≥ 100mL/m^2^ (female); right ventricular ejection fraction ≤ 40%. The minor criteria are the motion changes already described as major criteria accompanied by right ventricular end-diastolic index ≥ 100 and < 110 mL/m^2^ (male) and ≥ 90 and < 100mL/m^2^ (female), or ejection fraction > 40% and ≤ 45%.

The family history has the strength of a major criterion when arrhythmogenic right ventricular cardiomyopathy is diagnosed in first-degree relatives both by meeting the above-mentioned criteria and by a positive biopsy or postmortem examination, or even when mutations related to the development of cardiomyopathy are confirmed. The minor criteria are: suspected disease in a first-degree relative that cannot be confirmed; sudden death probably due to that cardiomyopathy in a first-degree relative before the age of 35 years; or confirmed diagnosis in a second-degree relative.

Regarding endomyocardial histopathology, the major criterion is less than 60% of the myocardial area occupied by cardiomyocytes at morphometric analysis (or <50% if estimated) with fibrous replacement of the right ventricular free wall in at least two endocardial samples, with or without fatty replacement. The minor criteria comprise the same changes described above and a residual myocyte rate between 60% and 75% by morphometric analysis (or between 50% and 65% if estimated).^5^

In our patient, we had no access to the original ECG tracing, and, thus, could not use it as a diagnostic method.

However, on the electrophysiological study, ventricular tachycardia with morphology of left bundle branch block and superior axis was triggered, a major criterion for that disease diagnosis.

The echocardiogram evidenced a dilated and hypokinetic right ventricle, but provided no detail to confirm the diagnosis.

Magnetic resonance imaging could not be performed because of the presence of the cardioverter defibrillator, implanted on the beginning of the clinical findings, when syncope was attributed to arrhythmia, which, along with left ventricular dysfunction, would be sequelae of a previous episode of myocarditis.

Although magnetic resonance imaging is considered the gold-standard test for the non-invasive diagnosis of that disease, the false-positive rate has been very high.^6^

The therapy of choice for patients with arrhythmogenic right ventricular cardiomyopathy is cardioverter defibrillator implantation, because neither the use of antiarrhythmic agents nor ablation on electrophysiological study proved to be reliable alternatives to reduce sudden death.^7^ (Desiderio Favarato, MD)

**Diagnostic hypothesis:** arrhythmogenic right ventricular cardiomyopathy. (Desiderio Favarato, MD)

## Anatomopathological examination

**Figure 1:**
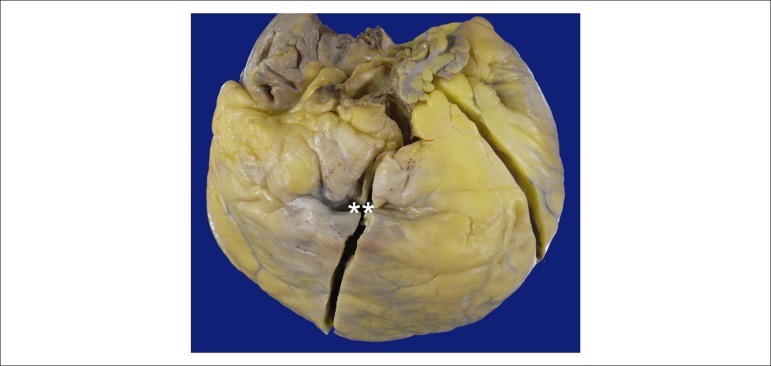
External view of the anterior face of the explanted heart. The epicardial fat is abundant, and a collapsed aneurysmal formation can be seen in the right ventricular outflow tract (asterisks).

**Figure 2:**
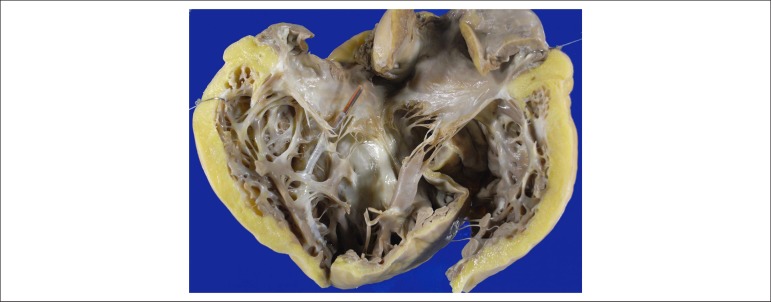
Right ventricular inlet. The cavity is markedly dilated, and the fatty infiltration of the compacted portion of the wall is evident, remaining only the trabecular musculature. Note the metallic lead of the cardioverter defibrillator in the ventricular apex.

**Figure 3:**
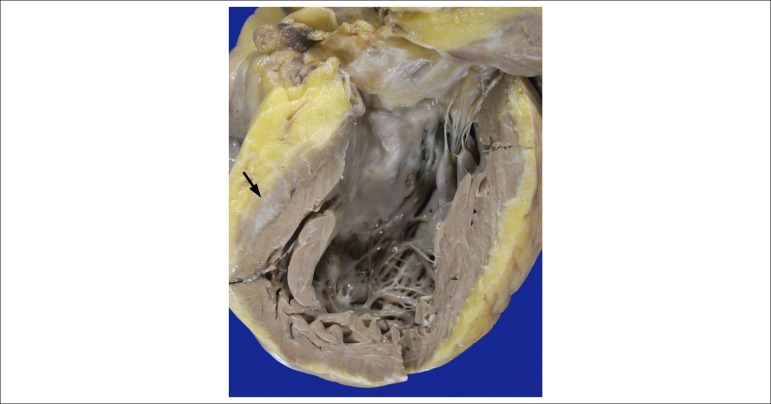
Left ventricular outflow tract. Note the moderate dilatation of the cavity, hypertrophy of the wall and area of subepicardial fibrosis (arrow).

**Figure 4:**
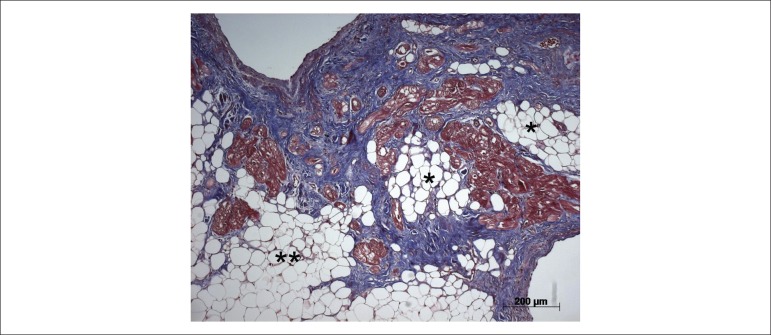
Histological section of the right ventricular inlet. The myocardium shows replacement with adipose cells (asterisks), and deposition of collagen (stained in blue) amid the cardiomyocytes (stained in red). Masson’s trichrome staining.

The explanted heart weighed 576 g, lacked a large part of the left atrium, was very enlarged and ball-shaped, and had abundant subepicardial fat. There was a bulging area of imprecise limits in the right ventricular outflow tract, corresponding to an aneurysmal formation (Figure 1). The right ventricle was markedly dilated and exhibited a metallic lead (cardioverter defibrillator lead) anchored in its apex, focally adhered to the free margin of the tricuspid valve. Extensive, diffuse fatty infiltration of the compacted portion of the right ventricular free wall in its inlet, apex and outflow tract was seen (Figure 2). The left ventricle showed moderate dilation and hypertrophy, with isolated foci of subepicardial fibrofatty infiltration (Figure 3). The microscopic exam confirmed the gross aspect of myocardial fatty infiltration, in addition to fibrosis (Figure 4). The most preserved areas of the myocardium showed hypertrophic cardiomyocytes, fibrosis foci, and interstitial mild lymphohistiocytic inflammatory infiltrate. The endocardium was thickened and whitish in the region of the aneurysmal formation of the right ventricular outflow tract. The heart valves and epicardial coronary arteries showed no abnormality. No cavitary thrombus was seen. (Luiz Alberto Benvenuti, MD)

**Anatomopathological diagnosis:** arrhythmogenic right ventricular cardiomyopathy. (Luiz Alberto Benvenuti, MD)

## Comments

The patient is a 36-year-old male with heart disease characterized by syncope episodes since the age of 20 years, cardiomegaly and ventricular dysfunction. He underwent cardioverter defibrillator implantation in 2001. His electrophysiological study evidenced scars and late potentials in several regions of the right ventricle, ventricular tachycardia being triggered during the exam in 2015. Because of progression of the ventricular dysfunction and heart failure, the patient underwent cardiac transplantation in 2016. The anatomopathological exam of the explanted heart revealed arrhythmogenic right ventricular cardiomyopathy, with marked fatty infiltration of the compacted portion of that ventricle, with aneurysmal formation in the outflow tract. In addition, there was impairment of the left ventricle, which showed foci of subepicardial fibrofatty infiltration. Arrhythmogenic right ventricular cardiomyopathy, also known as arrhythmogenic dysplasia, is a primary genetic cardiomyopathy, most commonly of dominant autosomal inheritance. Several mutations related to the disease have been identified, usually in genes encoding desmosomal proteins, the most known being the genes of desmoplakin and plakoglobin. It can be associated with Carvajal syndrome or Naxos disease (palmoplantar keratoderma/wooly hair). Arrhythmogenic right ventricular cardiomyopathy is a frequent cause of sudden death in young individuals, being the major cause of sudden death associated with sports activity in Italy.^8^ The disease can be restricted to the right ventricle, with severe arrhythmias, but, in the forms of progressive heart failure, as the present case, the left ventricle is commonly affected. Because both ventricles can be affected, many people advocate the use of the term 'arrhythmogenic cardiomyopathy'. The diagnosis of the disease is complex and multifactorial, and several elements should be considered, such as electrocardiographic changes, presence and type of arrhythmias, echocardiographic and magnetic resonance imaging changes, family history and even histological changes of the ventricular wall. Since 1994, and modified in 2010, there has been consensus about the diagnostic criteria, some considered major and others, minor.^9^ Although invasive, endomyocardial biopsy is indicated in selected cases to assess myocardial histology, and myocardial fibrofatty infiltration is considered a major criterion when the residual myocardium corresponds to less than 60%, and a minor criterion when the residual myocardium corresponds to 60% to 75% of the sample.^10^ (Luiz Alberto Benvenuti, MD)
